# Prevalence, Regional Variation, and Energy Cost of Ultra-Processed Foods Marketed to Children in Türkiye

**DOI:** 10.3390/nu18203444

**Published:** 2026-10-20

**Authors:** Gonca Yildirim, Esra Tansu Sariyer, Ekin Çevik, Betül Çiçek

**Affiliations:** 1 Faculty of Health Sciences, Nutrition and Dietetics, Toros University, Mersin 33140, Türkiye; 2 Hamidiye Faculty of Health Sciences, Nutrition and Dietetics, University of Health Sciences Türkiye, Istanbul 34668, Türkiye; esratansu.sariyer@sbu.edu.tr; 3 Faculty of Health Sciences, Nutrition and Dietetics, Üsküdar University, Istanbul 34768, Türkiye; ekin.cevik@uskudar.edu.tr; 4 Institute of Health Sciences, Nutrition and Dietetics, Erciyes University, Kayseri 38039, Türkiye; 5 Faculty of Health Sciences, Nutrition and Dietetics, Erciyes University, Kayseri 38260, Türkiye; bcicek@erciyes.edu.tr

**Keywords:** Ultra-processed foods, infant foods, child foods, NOVA classification, food prices, Türkiye

## Abstract

**Background:** Ultra-processed foods (UPFs) are becoming more common in children’s dietary habits, yet little is known about products marketed specifically to children in Türkiye. This research assessed the frequency, geographic differences, and energy-standardized cost of UPFs targeted at children. **Methods**: This cross-sectional study identified 340 food and beverage products aimed at children in six Turkish cities with varying regional income levels. We categorized items according to the NOVA framework. The prevalence of UPFs was analyzed by different cities, and the cost per 100 kcal was assessed between UPFs and non-UPFs. **Results**: Overall, 66.8% of products were classified as UPFs. Prevalence ranged from 53.3% to 83.7% across cities and differed significantly by product category. All milks, confections, and custards were UPFs of childhood. Most fruit and vegetable purees were not UPFs. The main markers of ultra-processing were added sugars, emulsifiers, and flavorants. The median cost for every 100 kcal was lower for UPFs than for non-UPFs (21.45 vs. 67.47 TRY, *p* < 0.001). After controlling for study city, non-UPFs had 2.87 times higher costs per 100 kcal than UPFs (95% CI: 2.27–3.62), but this association was not significant after further controlling for product category. **Conclusions**: UPFs were widely available in products marketed to children in Türkiye, with significant variation between product categories and regions. The lower cost per 100 kcal appeared to reflect differences in product composition across categories.

## 1. Introduction

The period from pregnancy to the end of a child’s second year, defined as the first 1000 days of life, is considered a critical period for growth, neurodevelopment, and the risk of chronic diseases in adulthood. Exclusive breastfeeding is the preferred and recommended form of feeding for infants during the first six months of life. After the sixth month, children should begin consuming safe and adequate complementary foods that can be prepared at home or commercially produced. Early nutrition, in particular, is a significant factor influencing eating behavior, food preferences, growth, and overall health, especially the risk of noncommunicable diseases [1,2,3].

In recent years, the commercial food market for infants and young children has grown rapidly both globally and in middle-income countries. Globally, the infant formula market rose from 9.6 billion U.S. dollars in 2010 to 17.9 billion U.S. dollars in 2022, and even higher market projections have been reported for 2030 [4]. Factors driving this growth include the increasing participation of women in the workforce, changes in household lifestyles, and rising income levels in developing economies [4]. During this period of change, there has been a noticeable increase in the introduction of food products containing ultra-processed food (UPFs), particularly in infant and baby formula, and this has become a cause for concern globally [2,4,5]. This is because a large portion of the products developed fall into the UPFs category according to the NOVA system, which classifies foods based on their sources and the purpose and extent of their processing. Monteiro et al., who developed the NOVA classification, note that UPFs are primarily formulations resulting from a series of industrial processes applied to ingredients that are typically used only in industrial settings [6]. Market surveys conducted in various countries indicate that the proportion of UPFs in commercial infant and complementary foods is at alarming levels [2,3,5]. Studies have shown that UPFs levels in infant formulas and complementary foods vary widely between 29 and 71%, with biscuits, cereal-based products, snacks, and finger foods showing particularly higher UPFs levels [1,2,3,7,8]. In addition, UPFs generally have an unbalanced and unhealthy nutrient composition because they are high in fat, sugar, salt, and energy [2,7]. Early consumption of these products has been found to be associated with chronic diseases, obesity, decreased dietary quality, and increased cardiometabolic risk in later life [3,9,10].

Food costs emerge as one of the key factors influencing consumers’ purchasing decisions and determining the dietary quality of groups in the low-to-middle socioeconomic bracket. Since healthy foods are more expensive than energy-dense, low-nutrient products, this increases the likelihood that low- and middle-income populations will purchase products with a lower or poorer nutrient composition [11,12]. In general, it is observed that the sales costs of baby food products, particularly organic ones, are higher and carry a premium ranging from 16% to 52% [11,13]. The existing literature indicates that the relationship between price and nutrient composition in infant foods is a universal issue; however, this relationship has not yet been examined in Türkiye, specifically concerning ultra-processed products. This study aims to fill a critical gap in both policy development and economic accessibility for parents by jointly assessing the prevalence and nutritional content of UPFs in the Turkish retail market.

## 2. Materials and Methods

This cross-sectional study was conducted to determine the prevalence of UPFs among foods and beverages marketed for children aged six months and older in the Turkish retail market and to examine differences in their prices across regions with different income distributions. The study comprised three main stages: (i) identification of eligible products and collection of price and nutritional composition data, (ii) classification of products according to the NOVA food processing classification system, and (iii) assessment of the associations between UPFs status, product characteristics, and price across the selected regions.

### 2.1. Product Definition, Study Setting, and Data Collection

Product and price data were collected online between February and April 2026. To account for regional socioeconomic variation, equivalized household disposable income reported in the 2024 Income Distribution Statistics of the Turkish Statistical Institute (TurkStat) was used [14]. NUTS Level-2 regions were ranked in ascending order according to equivalized household disposable income and divided into income quintiles. Within each quintile, a city with metropolitan municipality status was selected to represent the respective income level. The five selected cities were Ankara (Q1), Samsun (Q2), Manisa (Q3), Kastamonu (Q4), and Kocaeli (Q5), representing the five regional income quintiles, respectively. Istanbul was included separately because of its large population and distinct retail market, resulting in a total of six study cities. For consistency in reporting, the five cities were coded as C1–C5 according to their corresponding income quintiles.

Product and price information was obtained through the chain-market price database of the Scientific and Technological Research Council of Türkiye (TÜBİTAK), “marketfiyati.org” which provides access to product prices listed by major retail chains [15]. In each city, the municipality and governorate buildings were used as reference points to define the geographical search area. For each study city, products were identified through marketfiyati.org within a 10 km radius of the administrative centre. All listed products meeting the inclusion criteria were included, without restricting the search to specific retail chains. Products marketed specifically for children were identified using nine predefined search terms: “bebek” (baby), “bebe” (baby), “baby”, “mama” (baby food/formula), “püre” (purée), “devam” (follow-on), “çocuk” (child), “tamamlayıcı” (complementary), and “infant”. Products were considered eligible when their product name or marketing information indicated that they were intended for children. Product availability in physical stores was not independently verified.

Human milk substitutes were excluded because they are specifically formulated to meet the nutritional requirements of infants and may be necessary when breastfeeding is not possible or sufficient. As these products are inherently industrially formulated and generally classified as ultra-processed according to the NOVA system, their inclusion could disproportionately influence estimates of UPFs prevalence and limit comparability with other commercially available foods marketed to infants and children [6].

For each eligible product, the lowest regular (non-promotional) price available during the data collection period was recorded. Promotional prices and short-term campaign discounts were excluded. Unit prices were standardized as Turkish Lira per kilogram (TRY/kg) for solid products and Turkish Lira per liter (TRY/L) for liquid products. Nutritional composition and ingredient information were obtained from the product information available through the retail platform and manufacturers’ official sources. Energy contents were recorded per 100 g for solid products or per 100 mL for liquid products. Of the 340 products included in the study, 11 were excluded from the energy-standardized cost analyses because energy information was unavailable for 10 products, while one product (children’s water) had an energy value of zero. The excluded products comprised six desserts/puddings, two beverages, and one product each from the fruit/vegetable purée, cereal-based food, and biscuit/snack categories. Consequently, cost-per-100-kcal analyses were conducted using 329 products.

### 2.2. NOVA Classification According to Degree of Food Processing

Foods and beverages were classified according to the NOVA food processing classification system, which considers the nature, extent, and purpose of the physical, biological, and chemical processes applied during food production. NOVA classifies foods into four groups: unprocessed or minimally processed foods (NOVA 1), processed culinary ingredients (NOVA 2), processed foods (NOVA 3), and UPFs (NOVA 4) [6]. Products containing markers of ultra-processing, such as flavorings, emulsifiers, modified starches, non-sugar sweeteners, certain syrups or concentrates, and other ingredients characteristic of industrial formulations, were classified as NOVA 4 when consistent with the NOVA criteria. A free-text justification for each NOVA classification was recorded to facilitate subsequent verification.

For analytical purposes, NOVA 4 products were defined as UPFs, whereas NOVA 1–3 products were grouped as non-UPFs. The original four-category NOVA classification was retained for descriptive analyses.

### 2.3. Data Extraction and Coding

For each eligible food or beverage, information was collected using a standardized and piloted data extraction protocol and entered into a Microsoft Excel database. Data were systematically recorded by 5 senior Nutrition and Dietetics students and included: (i) city; (ii) product name; (iii) product category; (iv) standardized unit price (TRY/kg or TRY/L); (v) NOVA classification; (vi) markers of ultra-processing for products classified as NOVA 4; (vii) energy content per 100 g or 100 mL.

Products were assigned to one of 11 predefined categories: growing-up milks; biscuits, wafers and chips; cereals, porridges and cereal-based foods; fruit and vegetable purées; fruit juices, smoothies, teas and other beverages; meat- or fish-based meals; powdered beverages; desserts, puddings and dairy desserts; soups; yogurts; and other products. For NOVA 4 products, potential markers of ultra-processing were additionally coded as the presence of added sugar, added salt, non-sugar sweeteners, flavorings, emulsifiers, modified starches, concentrates or syrups, preservatives, and other relevant markers. The presence of free sugars was assessed according to the WHO definition [16].

### 2.4. Data Quality Control

To assess the accuracy and consistency of data extraction and classification, the senior author independently reassessed a random sample of 30 products. Agreement between the original data extraction and independent assessment was 100% for recorded price and NOVA classification and greater than 90% for nutritional composition data. Discrepancies were reviewed against the original product information and resolved by consensus.

### 2.5. Statistical Analysis

Categorical variables were summarized as frequencies and percentages, and continuous variables as medians with first and third quartiles (Q1–Q3). Distributional characteristics of continuous variables were evaluated using skewness and kurtosis values in accordance with the criteria proposed by Kim, 2013 [17]. Differences in the prevalence of UPFs across the six study cities were assessed using Pearson’s chi-square (χ^2^) test [17]. When the overall test was significant, pairwise comparisons between cities were performed with Bonferroni correction for multiple comparisons. Cost per 100 kcal was calculated from unit price and energy content and expressed in Turkish lira (TRY). Values were presented as median (Q1–Q3), and differences between UPFs and non-UPFs were assessed using the Mann–Whitney U test. Due to its skewed distribution, cost per 100 kcal was natural log-transformed for regression analyses. The association between UPFs status and cost per 100 kcal was examined using a generalized linear model with robust standard errors, accounting for study city. An interaction between UPFs status and study city was tested to determine whether the difference in cost per 100 kcal between UPFs and non-UPFs varied across cities. All analyses were performed using IBM SPSS Statistics for Windows, version 24.0 (IBM Corp., Armonk, NY, USA). A *p*-value < 0.05 was considered statistically significant [18].

## 3. Results

### Characteristics of the Sample

A total of 340 food products marketed for children were identified across the six study cities through marketfiyati.org. Istanbul accounted for the largest number of identified products (n = 89, 26.2%). Generally, cereals, porridges and cereal-based foods together with fruit and vegetable purées constituted the most common product group (n = 80, 23.5%), followed by growing-up milks (n = 72, 21.2%). Fourteen products (4.1%) were classified as “Other,” including pickled vegetables marketed for children, honey, and bars. The distribution of product categories across the six cities is presented in Table 1. Although 11 food categories were predefined, no eligible products were identified in the “meat or fish-based meals” and “powdered beverages” categories. Therefore, these categories were not included in Table 1 and Table 2.

According to the NOVA classification, 71 products (20.9%) were classified as NOVA 1, 8 (2.4%) as NOVA 2, 34 (10.0%) as NOVA 3, and 227 (66.8%) as NOVA 4. Overall, UPFs (NOVA 4) accounted for approximately two-thirds of all products (n = 227, 66.8%), while 113 products (33.2%) were classified as non-UPFs (NOVA 1–3).

The prevalence of UPFs varied across product categories. All growing-up milks (n = 72, 100.0%), desserts and puddings (n = 14, 100.0%), and products classified as “Other” (n = 14, 100.0%) were UPFs. Nearly all biscuits, wafers and chips were also classified as UPFs (n = 62, 98.4%). In contrast, all soups (n = 10, 100.0%) and yogurts (n = 3, 100.0%) were classified as non-UPFs. Fruit and vegetable purées were predominantly non-UPFs (n = 64, 80.0%), whereas cereals, porridges and cereal-based foods showed a more balanced distribution, with 47 products (58.8%) classified as UPFs and 33 (41.3%) as non-UPFs. Among fruit juices, smoothies, teas and other beverages, two of the four products (50.0%) were classified as UPFs (Table 2). Among UPFs, added sugar was the most frequently identified marker of ultra-processing (n = 131, 57.7%), followed by emulsifiers (n = 128, 56.4%) and flavorings (n = 110, 48.5%). Concentrates or syrups were identified in 69 products (30.4%), while other markers were present in 52 (22.9%) and added salt in 45 (19.8%). Sweeteners (n = 6, 2.6%) and preservatives (n = 2, 0.9%) were uncommon.

UPFs prevalence differed significantly across the six study cities, which represented different regional income levels (χ^2^(5) = 15.06, *p* = 0.010), ranging from 53.3% in C2 to 83.7% in C5. Pairwise comparisons identified a significant difference between C2 and C5 (adjusted *p* = 0.001) (Table 3).

UPFs, ultra-processed food. C1–C5 represent cities selected to represent different quintiles of equivalized household disposable income; Istanbul was included separately. Differences in UPFs prevalence across cities were assessed using Pearson’s chi-square test (χ^2^(5) = 15.06, *p* = 0.010). Bonferroni-adjusted pairwise comparisons identified a significant difference only between C2 and C5 (*p* = 0.001).

The median cost per 100 kcal was significantly lower for UPFs than for non-UPFs (21.45 [Q1–Q3: 11.85–68.33] vs. 67.47 [49.28–161.50] TRY; Mann–Whitney U = 6204.0, *p* < 0.001). In the primary regression model accounting for study city, the cost per 100 kcal of non-UPFs was 2.87 times that of UPFs (cost ratio = 2.87, 95% CI: 2.27–3.62, *p* < 0.001). The UPFs × study city interaction was not significant (Wald χ^2^(5) = 4.824, *p* = 0.438), indicating that the difference in cost per 100 kcal between UPFs and non-UPFs did not vary significantly across study cities. The sensitivity analysis additionally accounted for product category. However, after additional adjustment for product category, the association was no longer statistically significant (cost ratio = 1.07, 95% CI: 0.75–1.54, *p* = 0.707) (Table 4).

## 4. Discussion

This study analyzed the UPFs content of products aimed at children in the Turkish retail sector and revealed that approximately two-thirds of the products fall into the UPFs category. When prevalence was examined across product categories, all “growing-up milks,” as well as nearly all desserts, puddings, biscuits, wafers, and chips, were classified as UPFs. In contrast, fruit and vegetable purees consisted largely of non-UPFs products. The presence of UPFs in baby products varied significantly across the six cities studied (53.3–83.7%); however, pairwise comparisons revealed a significant difference between only two of the cities. Furthermore, the median cost per 100 kcal of UPFs was significantly lower than that of non-UPFs food products, and this difference persisted even after adjusting for city. However, after adjusting for product category, this relationship was no longer statistically significant. This indicates that the observed cost difference stems primarily from the product categories included in the UPFs and non-UPFs groups. The study’s findings demonstrate that UPFs constitute a significant component of foods marketed to children in Türkiye, and that there are notable differences in UPFs content and costs across product categories and regions.

In this study, approximately two-thirds (66.8%) of the food and beverages marketed for children aged six months and older in chain supermarkets in Türkiye were classified as UPFs. The marked differences in the prevalence of UPFs across product categories appear to be consistent with the literature. In a study conducted in Brazil, Rocha et al. reported that 79% of commercial foods intended for children under 36 months of age were ultra-processed, while only 4.2% were minimally processed. Furthermore, even when breast milk substitutes and follow-on formulas were excluded, all grain products and food supplements, as well as certain fruit and vegetable purees, were classified as UPFs [19]. Similarly, the proportion of UPFs among infant and toddler food products sold at the 10 largest supermarket chains in the United States was found to be 71% [7], a recent study conducted in Portugal revealed that, although the distribution varied by product group, 56.1% of commercial foods intended for children aged 0–36 months were UPFs and 10.2% were processed foods [1]. Similar to our findings, some studies have found that the UPFs rate varies by region within the same country. In Portugal, the proportion of UPFs among foods marketed to children aged 0–36 months was reported to be 56.1% in Porto [1] and 61.8% in the Lisbon Metropolitan Area [2]. In a recent study conducted in Turkey, products marketed to children aged 3 years and older in the province of Ankara were evaluated, and it was found that 93.4% of the foods contained UPFs [20]. A 2023 WHO report found that commercial foods for infants and young children in Ankara were being promoted extensively and inappropriately [21]. Our findings reveal that the prevalence of UPFs in the food market targeting infants and children is pronounced even during the complementary feeding period.

In this study, although UPFs rates varied significantly across product categories, the categories with the highest UPFs rates accounted for nearly half (44.1%) of all products. In other words, the overall prevalence of UPFs across products did not show a homogeneous distribution. While nearly all “growing-up milks,” desserts and puddings, and cookies, wafers, and chips were classified as UPFs, the majority of fruit and vegetable purees did not fall into the UPFs category. Breakfast cereals, porridges, and grain-based foods exhibited a more heterogeneous composition, with approximately 60% of them classified as UPFs. The classification of all growing-up milks as UPFs in this study is a notable and significant finding from a public health perspective. This may be due to the composition of follow-on formulas, which consist of milk powder, vegetable oils, caloric sweeteners, and additives. These products also help provide essential and non-essential nutrients to children [22].

In a study examining growth-stage milk products sold in supermarkets, Pries et al. found that 97% of the products contained at least one type of added sugar and that sugar levels varied significantly among the products [23]. A study conducted in Brazil also examined 12 products in the complementary food/growing-up milk category, all of which were classified as UPFs [19]. Similarly, in another study, 13 out of 14 complementary formula/growing-up milk products examined were classified as UPFs, while one was classified as a processed food [1]. WHO guidelines state that milk products specifically marketed for the nutrition of children up to 36 months of age, including follow-on formulas and “growing-up” milks, should be considered breast milk substitutes [24]. The European Society for Pediatric Gastroenterology, Hepatology, and Nutrition (ESPGHAN) Nutrition Committee also recommends that the routine use of these products is not necessary, but that they may be used as part of a strategy to increase intake of iron, vitamin D, and n-3 polyunsaturated fatty acids and to reduce protein intake compared to unfortified cow’s milk [25]. These recommendations underscore the importance of paediatric nurses, dietitians, and other healthcare professionals in providing evidence-based guidance to caregivers. In the absence of specific nutritional indications, counselling should prioritize age-appropriate dietary practices rather than the routine use of growing-up milks. Nevertheless, the classification of all growing-up milks as UPFs in our study should not be interpreted as indicating that these products have similar nutrient profiles or are necessarily nutritionally inadequate. Rather, this classification reflects the industrial processing criteria addressed by the NOVA system. Therefore, the degree of processing and nutritional composition should be considered as related but distinct dimensions when evaluating this product category.

The fact that UPFs are found in very high proportions not only in cookies, wafers, and chips but also in desserts and puddings is consistent with evidence showing that snack-type products marketed to infants and young children are particularly likely to be ultra-processed. In Europe, 62.2% of baby cookies and crackers and 42.1% of baby snacks are classified as UPFs [3]; in the United States, 85% of snacks and finger foods marketed to infants and young children, and 84% of confectionery products, were UPFs [7]. Similarly, 63% of commercial snacks produced for Australian infants and young children were UPFs; the majority of which had a sweet flavor profile (77%) and a texture that dissolves easily in the mouth (71%), and only 19% met all of the WHO’s relevant Nutrition and Labeling Profile Model requirements [26]. In Turkey, cakes and sweet cookies were among the most common packaged foods marketed to children aged 3 and older, and 93.4% of these products were UPFs [20]. On the other hand, marketing these products as infant or children’s foods may create the perception that they are healthier, which could pose health risks to children. Early dietary experiences and repeated exposure can shape food acceptance and subsequent eating habits. Furthermore, exposure to highly palatable and energy-dense foods during early childhood can influence food preferences later in life [27,28]. A study conducted in the United Kingdom found that UPFs accounted for 46.9% of energy intake among 21-month-old children, and by age 7, puddings and sweet breakfast cereals had become the primary sources of UPFs. Furthermore, it was observed that children in the highest UPFs consumption quartile during early childhood were 9.4 times more likely to remain in the highest quartile during middle childhood [29]. However, given the small number of desserts and puddings in our sample (n = 14), findings related to this category should be interpreted with caution.

In this study, an inverse relationship was observed between grain-based foods and fruit and vegetable purees. While 58.8% of cereals, porridges, and grain-based foods were classified as UPFs, only 20.0% of fruit and vegetable purees fell into the UPFs category. This difference is consistent with the literature indicating that grain-based products may be a significant source of UPFs exposure during early childhood. When data from 14,558 children under the age of 5 were analyzed in the Brazilian National Child Nutrition Survey, infant cereals were among the most frequently consumed groups of UPFs, with 29.4% of children reported to consume them [30]. Furthermore, similar to the data in our study, a study evaluating products in Europe found that 57.1% of cereal products for infants were UPFs, while only 18.2% of fruit products were classified as UPFs [3]. This difference is likely related to product formulations. This is because grain-based products often contain additives such as added sugar, maltodextrin, and flavorings, whereas purees generally contain only a limited number of additives or none at all [6]. Nevertheless, it should not be forgotten that when evaluating products, the focus should be on nutrient content rather than solely on the NOVA classification. This situation underscores the need for authorities to develop systems that evaluate both NOVA-based assessments and nutrient composition. In contrast, in this study, half of the fruit juices, smoothies, teas, and other beverages were classified as UPFs, while none of the soups or yogurts fell into the UPFs category. Contrary to these results, data in the literature indicate that yogurt products marketed to children fall within the UPFs subcategories [29,30]. The results from our study may be due to the small sample size in our categories of yogurt, fruit juices, smoothies, and teas. Therefore, the findings should be interpreted with caution, and the limitations of the sample should not be overlooked.

In this study, added sugar emerged as the most frequently detected indicator of UPFs in products, followed by emulsifiers and flavorings. Similarly, another study conducted in Türkiye examined commercial infant and children’s food products and found that nearly half of the products contained added sugar or sweeteners. Furthermore, while added salt was present in 1 out of every 10 products, 40% of the products exceeded the WHO European sodium standards [31]. Similar concerns have been reported in studies conducted in European countries. An analysis by Grammatikaki et al. of 3427 commercially available infant foods from 27 European countries revealed that 38.5% of the products contained at least one ingredient contributing added sugar; approximately 10% contained added sugar, and nearly one-quarter contained free sugars [3]. While such ingredients make products on store shelves appear healthier to caregivers, they also lead to early exposure to free sugars and sweet tastes. The WHO European Region’s Nutrient and Promotion Profile Model emphasizes the need to limit the use of sugar and sweeteners in commercial foods intended for children aged 6–36 months, to develop policies to this end, and to ensure that consumers are not misled by product labels [32]. Furthermore, the high levels of emulsifiers and flavorings identified in infant and child products in this study are consistent with findings in the literature [7]. In a study by Barnard et al. examining commercially available complementary food products suitable for children aged 6–23 months, it was found that nearly half (48.6%) of the infant beverages labeled as suitable for use starting at 6 months contained flavorings [33]. Although the use of emulsifiers in foods is permitted, while evidence showing their adverse effects on the gut microbiota, particularly in adults, is increasing, studies on their effects in children and infants remain limited [34,35]. It should not be forgotten that exposure to these additives during early childhood and infancy, one of the most critical periods for the development of the gut microbiota and health, may have potential effects [36,37]. In contrast, sugar-free sweeteners and preservatives are rarely found. In this context, the UPFs status of these products may stem more from added sugars and ingredients used to alter flavor, texture, and formulation than from these additives alone.

The prevalence of UPFs in the six cities assessed in the study varied greatly, from 53.3% to 83.7%; however, pairwise comparisons demonstrated a statistically significant difference only between cities C2 and C5. There was no apparent income level trend to this distribution. Moreover, despite having the highest equivalized household disposable income among the NUTS-2 regions of Türkiye, the presence of UPFs in the city was recorded as 66.3%, which is a moderate figure in the research sample. This points out that the presence of UPFs targeted at children is not driven by regional income levels alone. Research in the literature indicates that the issue has a more complex structure. Studies on commercial products aimed at infants and young children reveal that socioeconomic inequalities are not unidimensional. A Brazilian study examining commercial products for children 0–36 months of age found that the proportion of UPFs was similar between affluent and impoverished areas, but the type of retail outlet selling these products varied across areas [19]. In Portugal, De Araújo et al. found differences in the distribution of processing levels for commercial children’s products in neighborhoods with different income levels, but UPFs were ubiquitous in both regions [1]. A comprehensive survey of Brazil’s food environment showed that UPFs availability varied by retailer type and neighborhood sociodemographic characteristics, with supermarkets having the highest availability, especially in poorer neighborhoods [38]. Furthermore, the greater availability of processed and UPFs ready to eat in neighborhoods has been associated with higher consumption of these foods and a lower consumption of unprocessed or minimally processed foods by children [39]. Similarly, a large study of 492,630 children showed that the children’s UPFs consumption patterns varied from region to region [40]. Recent research about school food environments has shown that increased access to UPFs is associated with increased consumption, highlighting the influence of local food environments on dietary exposure [41]. Overall, the results suggest that the differences between cities observed in this study may reflect differences in retail structures, local product diversity, distribution systems, and general food environment characteristics rather than income. Such geographical differences are important, as differences in availability can lead to inequalities in children’s access to and consumption of UPFs.

Another significant finding of this study was that the cost per 100 kcal of UPFs was substantially lower than that of minimally processed foods. The median cost per 100 kcal was determined to be 21.45 TL for UPFs and 67.47 TL for minimally processed foods. In a model adjusted for the city where the study was conducted, the cost per 100 kcal of minimally processed foods was found to be 2.87 times higher than that of UPFs. This finding aligns with previous evidence indicating that UPFs generally provide energy at a lower cost. A US-based study determined the cost of UPFs to be 0.55 USD per 100 kcal, whereas the cost of unprocessed foods was 1.45 USD per 100 kcal; it also reported that UPFs have a higher energy density [42]. In Belgium, a high consumption share of UPFs was associated with lower costs, while minimally processed foods increased the total cost of the diet [43]. Similarly, in the Netherlands, costs associated with UPFs were found to be lower than those associated with minimally processed foods [44]. These results are consistent with the general literature indicating that energy-dense foods, such as refined grains, fats, and sweets, provide energy at a relatively low cost, whereas fruits, vegetables, and other foods with lower energy density tend to be more expensive per unit of energy [45]. Longitudinal pricing data from Brazil suggest that these price disparities may widen over time. Maia et al. reported that the prices of unprocessed or minimally processed foods have risen, while the relative prices of UPFs have declined. This trend has raised concerns that access to healthier dietary choices may become increasingly difficult [46]. Furthermore, the association identified in this study lost statistical significance after adjusting for product category. This indicates that the apparent energy-cost advantage of UPFs in foods marketed to children stems primarily from the types of products included in the UPFs and non-UPFs categories, rather than from the level of processing itself. This interpretation seems particularly plausible given the substantial variation in UPFs prevalence across the product categories in our sample. Consequently, affordability estimates based solely on the NOVA classification may mask the influence of product category and energy density on observed price differences. The absence of a significant interaction between UPFs status and city indicates that the relative difference in energy costs between UPFs and non-UPFs foods is remarkably similar across the six cities studied.

This study has several limitations. Product and pricing information was obtained from an online retail price database and may not reflect the full range of products available in other retail settings. Data collection was restricted to a 10 km radius around the administrative centre of each city, and differences in the retail chains represented within these areas may have influenced product coverage and prices. The availability of listed products in physical stores was not independently verified. Furthermore, the number of eligible products varied across cities (41–89), potentially introducing sampling bias and affecting comparisons between cities. Although the six cities were selected to represent different income levels, the sample was not nationally representative. The study assessed products listed in the database rather than actual purchasing or consumption patterns. Some food categories, particularly yoghurt (n = 3) and juices/beverages (n = 4), contained few products, limiting the reliability of category-specific comparisons. These categories were retained separately because of their distinct product characteristics. NOVA classification relied on the available ingredient information. Additionally, cost per 100 kcal does not account for serving size, household purchasing power, or overall affordability. Price changes associated with inflation during the February–April 2026 data collection period and differences in household disposable income across cities were not accounted for. Therefore, energy-standardized costs should not be interpreted as direct measures of household affordability.

Also, the exclusion of 11 products with missing or zero energy values from the energy-standardized cost analyses may have introduced selection bias, particularly because these exclusions were unevenly distributed across food subcategories, potentially affecting the regression estimates.

Despite these limitations, the study provides a comparative assessment of food products marketed to children across cities representing different income levels, using a consistent NOVA classification and energy-standardized cost measure.

## 5. Conclusions

Overall, around two-thirds of food and drink products marketed to children were ultra-processed, with wide variations across product categories and research locations. UPFs were found to have higher levels of sugar, emulsifying agents, and flavor enhancers. Although UPFs had a lower cost per 100 kcal, this difference disappeared after controlling for product category. These findings show the widespread presence of UPFs in the Turkish children’s food market and require ongoing surveillance of their contents, availability, and pricing. Although UPFs had a lower cost per 100 kcal than non-UPFs in the observed retail product mix, this difference was no longer statistically significant after adjustment for product category. These findings describe energy-standardized price differences rather than household affordability, which also depends on disposable income, purchasing power, and portion size.

## Figures and Tables

**Table 1:** Distribution of product subcategories by city.

Product Subcategory	City 1	City 2	City 3	City 4	City 5	Istanbul
Growing-Up Milks (n: 72, 21.2%)	19.1	17.3	17.8	17.1	16.3	31.5
Biscuits, Wafers and Chips (n: 63, 18.5%)	27.7	22.7	13.3	26.8	18.6	9.0
Cereals, Porridges and Cereal-Based Foods (n: 80, 23.5%)	19.1	42.7	15.6	14.6	23.3	18.0
Fruit and Vegetable Purées (n: 80, 23.5%)	19.1	10.7	28.9	34.1	30.2	25.8
Fruit Juices, Smoothies, Teas and Other Beverages (n: 4, 1.2%)	0.0	1.3	0.0	0.0	0.0	3.4
Desserts and Puddings (n: 14, 4.1%)	6.4	1.3	6.7	0.0	7.0	4.5
Soups (n: 10, 2.9%)	4.3	1.3	4.4	4.9	0.0	3.4
Yogurts (n: 3, 0.9%)	2.1	0.0	4.4	0.0	0.0	0.0
Other (n: 14, 4.1%)	2.1	2.7	8.9	2.4	4.7	4.5

Values are presented as percentages (%). Cell shading represents the relative percentage of products within each city, with darker shading indicating a higher proportion. City1–City5 indicate cities representing different quintiles of equivalized household disposable income; Istanbul was included separately.

**Table 2:** Distribution of products according to product category and NOVA classification.

Product Subcategory	Total, n (%)	UPFs, n (%)	Non-UPFs, n (%)
Growing-Up Milks	72 (21.2)	72 (100.0)	0 (0.0)
Biscuits, Wafers and Chips	63 (18.5)	62 (98.4)	1 (1.6)
Cereals, Porridges and Cereal-Based Foods	80 (23.5)	47 (58.8)	33 (41.3)
Fruit and Vegetable Purées	80 (23.5)	16 (20.0)	64 (80.0)
Fruit Juices, Smoothies, Teas and Other Beverages	4 (1.2)	2 (50.0)	2 (50.0)
Desserts and Puddings	14 (4.1)	14 (100.0)	0 (0.0)
Soups	10 (2.9)	0 (0.0)	10 (100.0)
Yogurts	3 (0.9)	0 (0.0)	3 (100.0)
Other	14 (4.1)	14 (100.0)	0 (0.0)
Total	340 (100.0)	227 (66.8)	113 (33.2)

**Table 3:** Prevalence of UPFs across study cities representing different income quintiles.

City	Total, n	UPFs, n (%)	Non-UPFs, n (%)
C1	47	35 (74.5)	12 (25.5)
C2	75	40 (53.3)	35 (46.7)
C3	45	33 (73.3)	12 (26.7)
C4	41	24 (58.5)	17 (41.5)
C5	43	36 (83.7)	7 (16.3)
Istanbul	89	59 (66.3)	30 (33.7)
Total	340	227 (66.8)	113 (33.2)

**Table 4:** Cost per 100 kcal according to UPFs status. (**A**) Cost per 100 kcal by NOVA classification. (**B**) Adjusted robust model for cost per 100 kcal.

**(A)**					
**NOVA Classification**	**n**	**Cost per 100 kcal, Median (Q1–Q3), TRY**			
UPFs	219	21.45 (11.85–68.33)			
Non-UPFs	110	67.47 (49.28–161.50)			
Total	329	36.11 (15.67–89.95)			
**(B)**					
**Analysis**	**β**	**Cost Ratio (95% CI)**	**Wald χ^2^**	***df***	***p***
Primary model: Non-UPFs vs. UPFs ^a^	1.053	2.87 (2.27–3.62)	77.424	1	<0.001
UPF × study city ^b^	—	—	4.824	5	0.438
Sensitivity model: Non-UPFs vs. UPFs ^c^	0.069	1.07 (0.75–1.54)	0.141	1	0.707

Cost per 100 kcal could be calculated for 329 of 340 products with available non-zero energy values. TRY, Turkish lira. Cost per 100 kcal was log-transformed for regression analyses. Cost ratios (95% CIs) were obtained by exponentiating the model coefficients. ^a^ Primary model included UPFs status and study city. ^b^ The UPFs × study city interaction represents the overall interaction across the six study cities. ^c^ Sensitivity model additionally included product category. UPFs, Ultra-processed foods.

## Data Availability

The data used in this study were obtained from publicly accessible sources, including Market Fiyatı (https://marketfiyati.org.tr/; (accessed on 15 March 2026) for product and pricing information and the Turkish Statistical Institute (TurkStat; https://veriportali.tuik.gov.tr/en; accessed on 1 March 2026) for regional income data. The compiled dataset used for the analyses is available from the corresponding author upon reasonable request.

## Abbreviations

The following abbreviations are used in this manuscript:

UPFs	Ultra-processed foods
